# Nd_2_(WO_4_)_3_

**DOI:** 10.1107/S1600536809018108

**Published:** 2009-05-23

**Authors:** Matthias Weil, Berthold Stöger, Lyubomir Aleksandrov

**Affiliations:** a Institute for Chemical Technologies and Analytics, Division of Structural Chemistry, Vienna University of Technology, Getreidemarkt 9/164-SC, A-1060 Vienna, Austria; b Institute of General and Inorganic Chemistry, Bulgarian Academy of Sciences, Acad. Georgi Bonchev Str. Bld.11, 1113 Sofia, Bulgaria

## Abstract

The title compound, dineodymium(III) tris­[tungstate(VI)], is a member of the Eu_2_(WO_4_)_3_ structure family and crystallizes isotypically with other rare earth tungstates and molybdates of this formula type. The structure is a derivative of the scheelite (CaWO_4_) structure and can be considered as an ordered defect variant with a threefold scheelite supercell and one rare earth (*RE*) site unoccupied. The Nd^3+^ cations are coordinated by eight O atoms in form of a distorted bicapped trigonal prism. The two unique W cations are tetra­hedrally surrounded by O atoms. One WO_4_ tetra­hedron has 2 symmetry and is relatively undistorted whereas the other tetra­hedron differs considerably from an ideal geometry. This is caused by an additional remote O atom at a distance of 2.149 (4) Å. The resulting WO_4 + 1_ polyhedra form W_2_O_8_ dimers through edge-sharing. Together with the WO_4_ and NdO_8_ units, the three-dimensional set-up is accomplished.

## Related literature

The crystal structure determination of scheelite was reported by Dickinson (1920 ▶). For a previous investigation of the structure of Nd_2_(WO_4_)_3_, see: Nassau *et al.* (1969 ▶). Isotypic (*RE*)_2_(WO_4_)_3_ structures have been reported by Templeton & Zalkin (1963 ▶) for *RE* = Eu; Gärtner *et al.* (1994 ▶) for La; Rong *et al.* (2003 ▶) for Dy, Gressling & Müller-Buschbaum (1995 ▶) for Ce. For the role of the crystal structure on the thermal expansion of (*RE*)_2_(WO_4_)_3_ compounds, see: Sumithra & Umarji (2004 ▶). For data standardization, see: Gelato & Parthé (1987 ▶).

## Experimental

### 

#### Crystal data

Nd_2_(WO_4_)_3_
*M* *_r_* = 1032.03
Monoclinic, 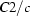

*a* = 7.7589 (12) Å
*b* = 11.597 (2) Å
*c* = 11.516 (2) Å
β = 109.561 (14)°
*V* = 976.4 (3) Å^3^
*Z* = 4
Mo *K*α radiation
μ = 45.72 mm^−1^
*T* = 293 K
0.12 × 0.10 × 0.08 mm

#### Data collection

Enraf–Nonius CAD-4 diffractometer
Absorption correction: numerical (*HABITUS*; Herrendorf, 1997 ▶) *T*_min_ = 0.033, *T*_max_ = 0.119
8392 measured reflections
2149 independent reflections
1830 reflections with *I* > 2σ(*I*)
*R*_int_ = 0.082
3 standard reflections frequency: 120 min intensity decay: none

#### Refinement

*R*[*F* ^2^ > 2σ(*F* ^2^)] = 0.025
*wR*(*F* ^2^) = 0.063
*S* = 1.09
2149 reflections
79 parameters
Δρ_max_ = 2.79 e Å^−3^
Δρ_min_ = −5.24 e Å^−3^

### 

Data collection: *CAD-4 Software* (Enraf–Nonius, 1989 ▶); cell refinement: *CAD-4 Software*; data reduction: *HELENA* implemented in *PLATON* (Spek, 2009 ▶); program(s) used to solve structure: *SHELXS97* (Sheldrick, 2008 ▶); program(s) used to refine structure: *SHELXL97* (Sheldrick, 2008 ▶); molecular graphics: *ATOMS* (Dowty, 2006 ▶); software used to prepare material for publication: *SHELXL97*.

## Supplementary Material

Crystal structure: contains datablocks I, global. DOI: 10.1107/S1600536809018108/si2175sup1.cif

Structure factors: contains datablocks I. DOI: 10.1107/S1600536809018108/si2175Isup2.hkl

Additional supplementary materials:  crystallographic information; 3D view; checkCIF report

## Figures and Tables

**Table 1:** Selected bond lengths (Å)

Nd—O4^i^	2.387 (4)
Nd—O2^i^	2.391 (4)
Nd—O3^i^	2.427 (5)
Nd—O6^ii^	2.433 (4)
Nd—O1^iii^	2.487 (4)
Nd—O5^iv^	2.488 (4)
Nd—O1	2.495 (4)
Nd—O2	2.497 (4)
W1—O5^v^	1.741 (4)
W1—O4	1.771 (4)
W1—O2^vi^	1.838 (4)
W1—O6	1.881 (4)
W2—O3	1.754 (4)
W2—O1	1.808 (4)

Symmetry codes: (i) 
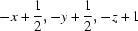
; (ii) 
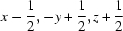
; (iii) 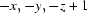; (iv) 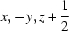; (v) 
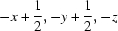
; (vi) 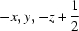.

**Fig. 1.**
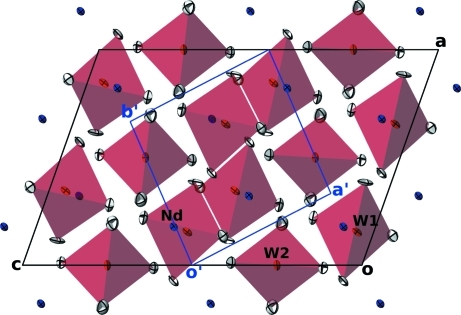
The crystal structure of Nd2(WO4)3 in a projection along the b- axis, drawn with displacement ellipsoids at the 74% probability level. Nd atoms are given in blue, W atoms in red and O atoms in white. WO4 units are given as red tetrahedra. Nd—O bonds have been omitted for clarity. The unit cell of the (distorted) scheelite substructure is indicated. with blue lines.

## supplementary crystallographic information

### Comment

Rare earth tungstates with formula (*RE*)_2_(WO_4_)_3_ are interesting materials due to their negative thermal expansion behaviour (Sumithra & Umarji, 2004). Therefore detailed structural data are required for a better understanding and a quantification of these effects. For a number of (*RE*)_2_(WO_4_)_3_ structures single-crystal data were already published: *RE* = Eu (Templeton & Zalkin, 1963); La (Gärtner *et al.*, 1994); Dy (Rong *et al.*, 2003); Ce (Gressling & Müller-Buschbaum, 1995). Preparation and an investigation of the crystal structure and the thermal behaviour of the Nd member were also reported some time ago by Nassau *et al.* (1969), including indication of a phase transition at 1318 K. However, the structural characterization of both low- and high-temperature phases remained preliminary. Although we tried to isolate the proposed high-temperature phase of Nd_2_(WO_4_)_3_ by rapid quenching of the sample from above the transition temperature, we could obtain only the low-temperature polymorph. Here we report the details of the corresponding α-Nd_2_(WO_4_)_3_ structure.

The above mentioned (*RE*)_2_(WO_4_)_3_ compounds are isotypic with Nd_2_(WO_4_)_3_ and can be considered as a derivative of the scheelite (CaWO_4_) structure (Dickinson, 1920). The (*RE*)_2_(WO_4_)_3_ structure is an ordered defect variant with a threefold scheelite supercell. The matrix that relates the scheelite structure with the (*RE*)_2_(WO_4_)_3_ structure is (110; 001; 210) (Fig. 1). In the "Ca_3_(WO_4_)_3_" supercell structure one rare earth site is unoccupied, thus leading to the formula (*RE*)_2_(WO_4_)_3_ and distortions of the coordination polyhedra as a consequence.

The Nd^3+^ cations are coordinated by eight oxygen atoms in form of a distorted bi-capped trigonal prism. The Nd—O distances range from 2.387 (4) to 2.497 (4), conform with the *RE*—O distances in the isotypic compounds. The W atoms are tetrahedrally surrounded by oxygen atoms. The W2 atom lies at Wyckoff site 4*e* with site symmetry 2 and W—O distances between 1.754 (4) and 1.808 (4) Å. The W1 atom is on a general position with similar distances between 1.741 (4) and 1.881 (4) Å. An additional O atom lies 2.149 (4) Å from W1, resulting in an overall '4 + 1' coordination for the W1 atom. These W(1)O_4 + 1_ polyhedra share edges, thus forming W(1)_2_O_8_ dimers. Together with the W(2)O_4_ and NdO_8_ units, the three-dimensional structure is assembled (Fig. 1).

### Experimental

Single crystals of the title compound were obtained from the melt. Analytical grade starting materials Nd_2_O_3_ (Fluka, 99.9%) and WO_3_ (Aldrich, 99.5%) were mixed and heated under atmospheric conditions in a platinum crucible to 1473 K. Then the furnace was slowly cooled to 1273 K during 24 h, held at that temperature for 5 h and then cooled to 1173 K during 30 h. Then the crucible was taken from the furnace and was quenched in water. Light-purple crystals of the title compound suitable for X-ray diffraction studies were broken from a large chunk by gentle crushing between two glass slides.

### Refinement

The highest peak is 0.76 Å from W2 and the deepest hole 0.69 Å from the same atom. Finally, structure data were standardized with the program *STRUCTURETIDY* (Gelato & Parthé, 1987).

### Crystal data

**Table:**

Nd_2_(WO_4_)_3_	*F*(000) = 1752
*M**_r_* = 1032.03	*D*_x_ = 7.021 Mg m^−^^3^
Monoclinic, *C*2/*c*	Mo *K*α radiation, λ = 0.71073 Å
Hall symbol: -C 2yc	Cell parameters from 25 reflections
*a* = 7.7589 (12) Å	θ = 10.5–16.9°
*b* = 11.597 (2) Å	µ = 45.72 mm^−^^1^
*c* = 11.516 (2) Å	*T* = 293 K
β = 109.561 (14)°	Block, light-purple
*V* = 976.4 (3) Å^3^	0.12 × 0.10 × 0.08 mm
*Z* = 4	

### Data collection

**Table:**

Enraf–Nonius CAD-4 diffractometer	1830 reflections with *I* > 2σ(*I*)
Radiation source: fine-focus sealed tube	*R*_int_ = 0.082
graphite	θ_max_ = 35.0°, θ_min_ = 3.3°
ω/2θ scans	*h* = −12→12
Absorption correction: numerical (*HABITUS*; Herrendorf, 1997)	*k* = −18→18
*T*_min_ = 0.033, *T*_max_ = 0.119	*l* = −18→18
8392 measured reflections	3 standard reflections every 120 min
2149 independent reflections	intensity decay: none

### Refinement

**Table:**

Refinement on *F*^2^	Primary atom site location: structure-invariant direct methods
Least-squares matrix: full	Secondary atom site location: difference Fourier map
*R*[*F*^2^ > 2σ(*F*^2^)] = 0.025	*w* = 1/[σ^2^(*F*_o_^2^) + (0.0271*P*)^2^] where *P* = (*F*_o_^2^ + 2*F*_c_^2^)/3
*wR*(*F*^2^) = 0.063	(Δ/σ)_max_ = 0.001
*S* = 1.09	Δρ_max_ = 2.79 e Å^−^^3^
2149 reflections	Δρ_min_ = −5.24 e Å^−^^3^
79 parameters	Extinction correction: *SHELXL97* (Sheldrick, 2008), Fc^*^=kFc[1+0.001xFc^2^λ^3^/sin(2θ)]^-1/4^
0 restraints	Extinction coefficient: 0.00516 (13)

### Special details

**Table:**

Geometry. All e.s.d.'s (except the e.s.d. in the dihedral angle between two l.s. planes) are estimated using the full covariance matrix. The cell e.s.d.'s are taken into account individually in the estimation of e.s.d.'s in distances, angles and torsion angles; correlations between e.s.d.'s in cell parameters are only used when they are defined by crystal symmetry. An approximate (isotropic) treatment of cell e.s.d.'s is used for estimating e.s.d.'s involving l.s. planes.
Refinement. Refinement of *F*^2^ against ALL reflections. The weighted *R*-factor *wR* and goodness of fit *S* are based on *F*^2^, conventional *R*-factors *R* are based on *F*, with *F* set to zero for negative *F*^2^. The threshold expression of *F*^2^ > σ(*F*^2^) is used only for calculating *R*-factors(gt) *etc*. and is not relevant to the choice of reflections for refinement. *R*-factors based on *F*^2^ are statistically about twice as large as those based on *F*, and *R*- factors based on ALL data will be even larger.

### Fractional atomic coordinates and isotropic or equivalent isotropic displacement parameters (Å^2^)

**Table:**

	*x*	*y*	*z*	*U*_iso_*/*U*_eq_	
Nd	0.17830 (3)	0.12676 (2)	0.59450 (2)	0.00552 (7)	
W1	0.15461 (2)	0.355585 (18)	0.048983 (19)	0.00616 (7)	
W2	0.0000	0.11826 (3)	0.2500	0.00700 (7)	
O1	0.0124 (5)	0.0407 (4)	0.3888 (4)	0.0087 (6)	
O2	0.0717 (5)	0.3000 (4)	0.4608 (4)	0.0103 (7)	
O3	0.1944 (5)	0.2058 (4)	0.2795 (4)	0.0138 (8)	
O4	0.2205 (6)	0.4270 (4)	0.1932 (4)	0.0127 (7)	
O5	0.3630 (5)	0.0366 (4)	0.0596 (4)	0.0104 (7)	
O6	0.3846 (5)	0.2870 (4)	0.0765 (4)	0.0115 (7)	

### Atomic displacement parameters (Å^2^)

**Table:**

	*U*^11^	*U*^22^	*U*^33^	*U*^12^	*U*^13^	*U*^23^
Nd	0.00501 (10)	0.00694 (12)	0.00528 (12)	−0.00035 (7)	0.00262 (8)	0.00007 (8)
W1	0.00522 (9)	0.00682 (10)	0.00802 (10)	−0.00014 (6)	0.00430 (6)	0.00015 (6)
W2	0.00763 (11)	0.01014 (13)	0.00401 (12)	0.000	0.00297 (8)	0.000
O1	0.0106 (15)	0.0089 (16)	0.0069 (15)	−0.0007 (12)	0.0032 (12)	0.0005 (12)
O2	0.0089 (14)	0.0138 (18)	0.0111 (17)	0.0023 (13)	0.0074 (12)	0.0020 (14)
O3	0.0163 (17)	0.0133 (19)	0.0120 (18)	−0.0018 (14)	0.0050 (14)	−0.0037 (15)
O4	0.0142 (15)	0.0180 (19)	0.0057 (15)	0.0012 (15)	0.0031 (12)	−0.0004 (14)
O5	0.0110 (15)	0.0111 (17)	0.0102 (17)	−0.0023 (13)	0.0048 (13)	0.0025 (14)
O6	0.0035 (13)	0.0133 (18)	0.0178 (19)	−0.0002 (12)	0.0036 (13)	−0.0059 (15)

### Geometric parameters (Å, °)

**Table:**

Nd—O4^i^	2.387 (4)	W1—O5^v^	1.741 (4)
Nd—O2^i^	2.391 (4)	W1—O4	1.771 (4)
Nd—O3^i^	2.427 (5)	W1—O2^vi^	1.838 (4)
Nd—O6^ii^	2.433 (4)	W1—O6	1.881 (4)
Nd—O1^iii^	2.487 (4)	W1—O6^v^	2.149 (4)
Nd—O5^iv^	2.488 (4)	W2—O3	1.754 (4)
Nd—O1	2.495 (4)	W2—O3^vi^	1.754 (4)
Nd—O2	2.497 (4)	W2—O1^vi^	1.808 (4)
Nd—Nd^i^	3.9708 (7)	W2—O1	1.808 (4)
			
O4^i^—Nd—O2^i^	110.36 (14)	O5^v^—W1—O4	105.4 (2)
O4^i^—Nd—O3^i^	70.63 (16)	O5^v^—W1—O2^vi^	111.35 (18)
O2^i^—Nd—O3^i^	70.79 (14)	O4—W1—O2^vi^	101.06 (18)
O4^i^—Nd—O6^ii^	99.92 (14)	O5^v^—W1—O6	105.45 (19)
O2^i^—Nd—O6^ii^	130.65 (14)	O4—W1—O6	94.60 (19)
O3^i^—Nd—O6^ii^	84.51 (15)	O2^vi^—W1—O6	134.0 (2)
O4^i^—Nd—O1^iii^	73.56 (14)	O5^v^—W1—O6^v^	96.3 (2)
O2^i^—Nd—O1^iii^	148.71 (14)	O4—W1—O6^v^	157.2 (2)
O3^i^—Nd—O1^iii^	135.38 (14)	O2^vi^—W1—O6^v^	76.80 (16)
O6^ii^—Nd—O1^iii^	76.29 (14)	O6—W1—O6^v^	72.57 (17)
O4^i^—Nd—O5^iv^	87.53 (15)	O3—W2—O3^vi^	109.3 (3)
O2^i^—Nd—O5^iv^	70.42 (14)	O3—W2—O1^vi^	104.30 (18)
O3^i^—Nd—O5^iv^	124.37 (14)	O3^vi^—W2—O1^vi^	109.20 (19)
O6^ii^—Nd—O5^iv^	150.80 (14)	O3—W2—O1	109.20 (19)
O1^iii^—Nd—O5^iv^	78.93 (13)	O3^vi^—W2—O1	104.30 (18)
O4^i^—Nd—O1	139.13 (15)	O1^vi^—W2—O1	120.3 (3)
O2^i^—Nd—O1	95.50 (13)	W2—O1—Nd^iii^	126.85 (18)
O3^i^—Nd—O1	149.90 (14)	W2—O1—Nd	119.87 (19)
O6^ii^—Nd—O1	84.99 (14)	Nd^iii^—O1—Nd	111.72 (15)
O1^iii^—Nd—O1	68.28 (15)	W1^vi^—O2—Nd^i^	134.6 (2)
O5^iv^—Nd—O1	71.57 (13)	W1^vi^—O2—Nd	115.63 (18)
O4^i^—Nd—O2	140.26 (15)	Nd^i^—O2—Nd	108.61 (14)
O2^i^—Nd—O2	71.39 (14)	W2—O3—Nd^i^	136.9 (2)
O3^i^—Nd—O2	73.11 (15)	W1—O4—Nd^i^	137.0 (3)
O6^ii^—Nd—O2	60.61 (13)	W1^v^—O5—Nd^vii^	138.8 (2)
O1^iii^—Nd—O2	126.35 (12)	W1—O6—W1^v^	107.43 (17)
O5^iv^—Nd—O2	127.07 (13)	W1—O6—Nd^viii^	130.5 (2)
O1—Nd—O2	77.15 (13)	W1^v^—O6—Nd^viii^	106.94 (16)

Symmetry codes: (i) −*x*+1/2, −*y*+1/2, −*z*+1; (ii) *x*−1/2, −*y*+1/2, *z*+1/2; (iii) −*x*, −*y*, −*z*+1; (iv) *x*, −*y*, *z*+1/2; (v) −*x*+1/2, −*y*+1/2, −*z*; (vi) −*x*, *y*, −*z*+1/2; (vii) *x*, −*y*, *z*−1/2; (viii) *x*+1/2, −*y*+1/2, *z*−1/2.
